# Prevalence and determinants of depression in patients with epilepsy during the COVID-19 pandemic

**DOI:** 10.1186/s43045-022-00190-4

**Published:** 2022-03-15

**Authors:** Mohammad Gamal Sehlo, Wafaa Samir Mohamed, Usama Mahmoud Youssef, Shrouk Esam Lotfi, Ghada Mohamed Salah El-deen

**Affiliations:** 1 Psychiatry Department, Faculty of Medicine, Zagazig University, PO Box 44519, Zagazig, Egypt; 2 Neurology Department, Faculty of Medicine, Zagazig University, Zagazig, Egypt; 3 Neuropsychiatry Department, Abbaseya Hospital for Mental Illness, Cairo, Egypt

**Keywords:** COVID-19, Epilepsy, Depression

## Abstract

**Background:**

Epilepsy is one of the commonest and most serious neurological conditions. It is frequently associated with one or more medical or psychiatric comorbidities. Depression is one of the most common comorbidities. Patients with epilepsy (PWE) are expected to suffer from a high level of depression during the COVID-19 pandemic. This cross-sectional study was applied to 290 PWE. Data was collected by personal interviews with each patient using the Patient Health Questionnaire 9 (PHQ 9) scale for the diagnosis of depression and assessing its severity. We aimed to assess the prevalence and the risk factors of depression in PWE during the COVID-19 pandemic.

**Results:**

We found that 70.3% of PWE suffered from depression. Low financial status, refractory seizures, fear of infection and death by COVID-19, had close relatives died by COVID-19, had a sleep disturbance, a decreased family support, increased seizure rate during the pandemic, increased ER visits during the pandemic, lack of drug adherence, and decreased epilepsy-related follow-up visits during the pandemic were significantly associated with increased risk of depression in PWE during the pandemic.

**Conclusions:**

The COVID-19 pandemic has a serious effect on the psychological and physical well-being of PWE. There was an increased rate of depression during the COVID-19 pandemic in PWE with its subsequent burden on those patients. So, these patients are in a high need of care and support during the pandemic.

## Background

Epilepsy is one of the most frequent major brain disorders, affecting more than 70 million individuals worldwide [1]. Epilepsy contributes to 0.7% of the global burden of medical disorders [2].

Psychiatric comorbidities, such as mood, anxiety, and psychotic disorders, are common in epileptic patients, with rates that are often 2–3 times higher than in the general population [3]. Depression is the leading cause of poor quality of life in epileptic patients, and one of the most frequent psychiatric comorbidities [4].

The lifetime risk of depression in the general population is 15–18%, with almost one in five people experiencing one episode at some point in their lifetime [5]. Up to 30–50% of individuals with epilepsy have a depressive symptom, which often complicate seizure management and reduce overall quality of life [6].

The comorbidity of depression with epilepsy can be explained with several etiologies, including neurobiological mechanisms such as a hyperactive hypothalamic–pituitary–adrenal axis (HPA), neuroinflammatory mechanisms (IL-1b, IL-2, IL-6, interferon-g, and tumor necrosis factor-a) [7], neurotransmitter disturbance (serotonin and norepinephrine) [8], and anatomical abnormalities (prefrontal cortex and paralimbic structures) [9], also the social burden of the epilepsy itself on the patients.

The COVID-19 pandemic incorporates a lot of new stressful situations: loss of employment, the death of family members and colleagues, financial insecurity, and isolation from others, especially for people who live alone [10]. That can lead to a higher level of depression in PWE.

The frequency of depressive symptoms in the general population in the USA was found to be more than 3-fold higher during COVID-19 than before the pandemic. Individuals with fewer financial resources and more stressor exposure (such as job loss) reported more depressive symptoms [11].

Patients with epilepsy are expected to suffer from a high rate of depression during the COVID-19 pandemic. It was found that the prevalence of depression among patients with epilepsy (PWE) increased to 42.3% during the period of the pandemic [12].

The aims of this study were to assess the prevalence and the risk factors of depression in PWE during the COVID-19 pandemic. To our knowledge, this is the first study that had been conducted to assess depression among PWE in Egypt.

## Methods

### Participants

A sample of 290 consecutive patients diagnosed with epilepsy according to the International League Against Epilepsy (ILAE) classification 2017 were included in this cross-sectional study. The patients were recruited from the outpatient clinic and the inpatient ward of the Neurology Department, Zagazig University Hospital, Zagazig, Egypt, between August 2020 and September 2021.

Both male and female patients with an age range from 19 to 60 years were included in the study.

Exclusion criteria were patients with a past history of psychiatric illness, substance abuse, intellectual disability, chronic major medical disorders other than epilepsy, and previous or current infection with COVID-19.

### Measures

#### Sociodemographic and clinical data form

The sociodemographic and clinical data form is composed of questions related to personal and clinical characteristics of the patients and questions related to the COVID-19 pandemic. Including age, gender, marital status, employment status, number of children, educational degree, financial status, where and with whom the patient lives, family history of epilepsy, and psychiatric illness. Epilepsy-related data: type of seizures, response to antiepileptic drugs (AEDs) whether respondent or resistant, age of onset, time of seizure occurrence, number of drugs taken, rate of seizures before and during the pandemic, number of previous ER visits by a seizure, number of ER visits by a seizure during the pandemic, fear of having an uncontrolled seizure during the pandemic, drug adherence during the pandemic, routine follow-up during the pandemic. COVID-19-related data: closed people infection or death, following news about the pandemic, sleep disturbance during the pandemic, family support during the pandemic, job changes during the pandemic, financial changes during the pandemic, fear of job loss during the pandemic, fear of infection or death by COVID-19, fear of closed one’s infection or death by COVID-19, and sense of the end of the world.

#### Patient Health Questionnaire 9

The Patient Health Questionnaire (PHQ 9) will be used to assess depressive symptoms [13]. The PHQ 9 is a widely used measure for identifying depressive symptoms and diagnosing depressive disorders and has excellent psychometric properties when used in medical and psychiatric patients. The PHQ 9 incorporates DSM-IV diagnostic criteria for major depressive disorder, assessing the presence and severity of the nine primary symptoms of major depression. This enables not only the determination of the severity of depression but also the presence of depressive disorder. Scores of 5, 10, 15, and 20 represent mild, moderate, moderately severe, and severe depression respectively [14].

The validated Arabic version of the scale was used in this study [15].

### Statistical analysis

The data analysis and sample size calculations (with 80% power) were performed using the statistical package for social sciences (SPSS version 20). The categorical data were presented in the form of number and percentage. continuous data were expressed as mean ± SD (standard deviation) and median with the interquartile range (IQR). Chi-square was used as a test of significance of the differences among groups. Binary logistic regression analysis was used to assess the predictors of depression. A *P* value < 0.05 was considered to indicate statistical significance.

## Results

Table 1 shows that the age of the studied group ranged from 18 to 60 years with a mean of 33.69 years. Regarding sex, more than half of them were male (52.4%). About 50% of them live in urban areas; 55.9% live with their spouse and siblings. 56.2% of them were married. Regarding education and occupation, 54.1% had secondary education. 40.3% of them were working. Low financial status was found among 39% of them, while financial status was satisfying among 54.1% of them. Finally, 37.6% of them had no children and 39.6% had 1 to 2 children.

**Table 1:** Demographic characteristics of the studied group

Variable		(*n* = 290)	
*Age (years)*	Mean ± SD	33.69 ± 9.14	
	Range	18–60	
Variable		**No.**	**%**
*Sex*	Female	138	47.6
	Male	152	52.4
*Residence*	Urban	145	50
	Rural	145	50
*Live with*	Alone	12	4.1
	Spouse and siblings	162	55.9
	Parents	102	35.2
	Brother and sisters	10	3.4
	Sibling only	4	1.4
*Marital status*	Single	96	33.1
	Married	163	56.2
	Widow	6	2.1
	Divorced	25	8.6
*Education*	Illiterate	44	15.2
	Secondary	157	54.1
	University	80	27.6
	Post graduated	9	3.1
*Occupation*	Not working	173	59.7
	Working	117	40.3
*Financial status*	Low	113	39
	Satisfying	157	54.1
	High	20	6.9
*No. of children*	No	109	37.6
	1–2	115	39.6
	> 2	66	22.8

*SD* stander deviation

Table 2 shows that the median age of onset of epilepsy among the studied group was 17 years while duration was 14 years. About 21.4% of them had a positive family history of epilepsy, 2.8% had a positive family history of psychiatric disease, and 8.3% had a positive past history of psychiatric disease. The most frequent type of seizures found among the studied groups was generalized (43.4%); also 69.3% of the cases were responsive. Almost 89% of the studied group take more than one AEDs. Finally, 83.3% of the cases had seizures at any time.

**Table 2:** Clinical data of epilepsy among the studied group

Variable		(*n* = 290)	
*Age of onset (years)*	Median (IQR)	17 (10–24)	
*Duration (years))*	Median (IQR)	14 (7–23)	
Variable		**No.**	**%**
*Family history of epilepsy:*	Negative	228	78.6
	Positive	62	21.4
*Family history of psychiatric disease:*	Negative	282	97.2
	Positive	8	2.8
*Past history of psychiatric disease:*	Negative	266	91.7
	Positive	24	8.3
*Type of seizures*	Focal	96	33.2
	Generalized	126	43.4
	Focal with secondary generalization	68	23.4
*Response*	Respondent	201	69.3
	Refractory	89	30.7
*No. of AEDs*	1	33	11.4
	> 1	257	88.6
*Time of seizures*	Any time	243	83.8
	Day	26	9
	Night	21	7.2

*IQR* interquartile range

Table 3 shows that 28.3% of the studied group had moderately severe and 21% had severe depression according to the PHQ 9 score.

**Table 3:** Frequency of depression among the studied group

Variable		(*n* = 290)
*PHQ 9 score*	Mean ± SD	12.9 ± 7.38
	Median (IQR)	14 (4–19)
	Range	0–24
	No *N* (%)	86 (29.7%)
	Mild *N* (%)	18 (6.2%)
	Moderate	43 (14.8%)
	Moderately severe *N* (%)	82 (28.3%)
	Severe *N* (%)	61 (21%)

Table 4 shows that there was a statistically significant increase in the frequency of moderately severe to severe depression among persons who fear COVID-19 infection, fear of death from COVID-19 infection, had close people infected, had close people dead, had financial changes during the pandemic, had sleep disturbances during the pandemic, had decreased family support during the pandemic, and continuously follow news about the pandemic.

**Table 4:** The relationship between depression and COVID-19 among the studied group

Variable		*N*	None (*n* = 86)		Mild to moderate (*n* = 61)		Moderately severe to severe (*n* = 143)		*χ*^2^	*P*
			No.	%	No.	%	No.	%		
*Fear of infection by COVID-19*	No	136	51	37.5	24	17.6	61	44.9	**7.74**	0.02*
	Yes	154	35	22.7	37	24	82	53.2		
*Fear of death by COVID-19*	No	125	46	36.8	20	16	59	47.2	**6.63**	0.04*
	Yes	165	40	24.2	41	24.8	84	50.9		
*Closed people infection*	No	135	50	37	20	14.8	65	48.1	**9.36**	0.009*
	Yes	155	36	23.2	41	26.5	78	50.3		
*Closed people death*	No	272	81	29.8	61	22.4	130	47.8	**6.1**	0.04*
	Yes	18	5	27.8	0	0	13	72.2		
*Fear of job loss in the pandemic*	No	203	55	27.1	40	19.7	108	53.2	4.15	0.13 NS
	Yes	87	31	35.6	21	24.1	35	40.2		
*Job changes by the pandemic*	No	205	54	26.3	41	20	110	53.7	5.63	0.06 NS
	Yes	85	32	37.6	20	23.5	33	38.8		
*Financial changes in the pandemic*	No	62	36	58.1	6	9.7	20	32.3	**30.95**	< 0.001**
	Yes	228	50	21.9	55	24.1	123	53.9		
*Sleep disturbance during the pandemic*	No	213	77	36.2	48	22.5	88	41.3	**22.67**	< 0.001**
	Yes	77	9	11.7	13	16.9	55	71.4		
*Follow news about the pandemic*	Not follow	54	25	46.3	17	31.5	12	22.2	**27.95**	< 0.001**
	Low	73	22	30.1	8	11	43	58.9		
	Moderate	89	27	30.3	20	22.5	42	47.2		
	Continuous	74	12	16.2	16	21.6	46	62.2		

*χ*^*2*^ chi-square test, *NS* nonsignificant (*P* > 0.05); *significant (*P* < 0.05), **highly significant (*P* < 0.001)

Table 5 shows that there was a statistically significant increase in the frequency of moderately severe to severe depression among patients who had increased seizure rate, increased ER visits during the pandemic, patients who reported lack of drug adherence, and patients who reported decreased follow-up visits during the pandemic.

**Table 5:** The relationship between depression and epilepsy during the pandemic among the studied group

Variable		*N*	None (*n* = 86)		Mild to moderate (*n* = 61)		Moderately severe to severe (*n* = 143)		*χ*^2^	*P*
			No.	%	No.	%	No.	%		
*Rate of seizures after COVID-19:*	No change	233	86	36.9	40	17.2	107	45.9	**32.23**	< 0.001**
	Increase	57	0	0	21	36.8	36	63.2		
*Rate of ER visits after COVID-19:*	No change	109	38	34.9	35	32.1	36	33	**21.17**	< 0.001**
	Increase	118	48	26.5	26	14.4	107	59.1		
*Drug adherence during the pandemic*	No change	212	74	34.7	44	20.9	94	44.4	**7.68**	0.005*
	Lack	78	12	14.8	22	28.6	44	56.6		
*Routine follow-up during the pandemic*	No change	128	49	38.3	25	19.5	54	42.2	**8.35**	0.02*
	Lack	162	37	22.8	36	22.2	89	54.9		

*χ*^*2*^ chi-square test, *NS* nonsignificant (*P* > 0.05); *significant (*P* < 0.05), **highly significant (*P* < 0.001)

Table 6 shows that not working, low financial status, refractory seizures, fear of infection and death by COVID-19, had close people dead by COVID-19, had sleep disturbance, decreased family support, increased seizures rate during the pandemic, increased ER visits during the pandemic, lack of drug adherence, and decreased epilepsy-related follow-up visits increase the risk of depression by 5.83-, 11.90-, 14.60-, 4.99-, 3.65-, 12.71-, 3.95-, 7.10-, 5.76-, 4.3-, and 3.8-fold (odds ratio) respectively.

**Table 6:** Binary logistic regression analysis of the predictors of depression among the studied group

Variable	***B***	SE	Wald	***P***	OR	95% CI	
Age > 40	0.134	0.150	0.793	0.373	0.875	0.651	1.175
Female sex	1.774	1.016	2.456	0.060	0.062	0.009	0.457
Divorced	1.623	0.42	1.796	0.616	1.439	0.234	5.314.
Residence	0.776	0.562	1.905	0.168	2.173	0.722	6.544
Live alone	1.179	0.42	1.098	0.250	3.250	0.614	2.317
Illiterate	1.282	0.90	1.424	0.11	9.794	1.677	57.180
**Not working**	**8.671**	**2.059**	**17.736**	< 0.001**	**5.831**	**1.030**	**32.985**
> 2 children	1.041	0.848	1.508	0.219	2.832	0.538	14.909
**Low financial status**	**2.476**	**0.689**	**12.898**	< 0.001**	**11.896**	**3.080**	**45.949**
Positive family history of epilepsy	0.770	0.738	1.089	0.297	2.159	0.509	9.166
Positive family history of psychiatric disorder	3.389	1.716	3.901	0.048	29.624	1.026	855.25
Positive past history of psychiatric disorder	− 0.250	1.852	0.018	0.893	0.779	0.021	29.368
Age of onset < 17 years	0.242	0.160	2.303	0.129	0.785	0.574	1.073
Duration >14years	0.061	0.154	0.156	0.693	1.063	0.785	1.438
Generalized seizures	1.597	1.224	0.635	0.31	1.474	0.33	4.59
**Refractory**	**6.003**	**1.375**	**19.051**	< 0.001**	**14.60**	**7.31**	**59.93**
> 1 AEDs	1.109	0.917	1.464	0.226	3.032	0.503	18.279
Any time seizures	0.674	1.267	0.283	0.595	1.962	0.164	23.510
**Fear of infection by COVID-19**	**12.06**	**2.764**	**19.064**	< 0.001**	**5.06**	**2.12**	**20.001**
**Fear of death by COVID-19**	**5.608**	**2.102**	**5.85**	0.044*	**4.992**	**2.181**	**32.071**
Closed people infection	0.470	0.698	0.453	0.501	0.625	0.159	2.454
**Closed people death**	**7.046**	**3.132**	**1.060**	0.024*	**3.652**	**1.476**	**13.216**
Fear of job loss in the pandemic	1.177	3.435	1.757	0.100	1.347	0.894	3.217
Job changes by pandemic	3.801	2.335	2.649	0.104	2.738	0.460	14.349
Financial changes in the pandemic	− 0.344	1.102	0.097	0.755	0.709	0.082	6.154
**Sleep disturbance during the pandemic**	**4.287**	**1.092**	**15.414**	< 0.001**	**12.713**	**3.556**	**61.953**
**Decrease family support during the pandemic**	**3.057**	**1.706**	**10.006**	0.03*	**3.945**	**1.237**	**23.772**
Continuous Follow news about the pandemic	− 0.615	0.737	0.696	0.404	0.541	0.127	2.293
**Increase seizure rate**	**10.207**	**2.105**	**3.523**	< 0.001**	**7.098**	**2.438**	**26.762**
**Increase ER visit rate**	**8.566**	**1.676**	**3.701**	0.004*	**5.761**	**1.468**	**16.622**
**Lack of drug adherence during the pandemic**	**4.207**	**1.105**	**5.523**	0.02*	**4.270**	**2.438**	**22.627**
**Lack of routine follow-up during the pandemic**	**3.278**	**1.800**	**4.121**	0.03*	**3.757**	**1.158**	**16.635**

*OR* odds ratio, *CI* confidence interval; *significant (*P* < 0.05), **highly significant (*P* < 0.001)

## Discussion

To our knowledge, this is the first study that had been conducted to assess depression among PWE in Egypt. In our study, we assessed the prevalence and the determinants of depression in patients with epilepsy (PWE), during the COVID-19 pandemic.

In our study, the prevalence of depression in patients with epilepsy during the pandemic is 70.3%. 6.2% of the participants had mild depression, 14.8% had moderate depression, and 28.3% had moderately severe depression, while 21% had severe depression. Few studies have investigated the relationship between depression and COVID-19 in PWE, and all of them are consistent with our results about the increased prevalence of depression in those patients during the COVID-19 pandemic with varying percentages and this can be explained by the severe stress of the COVID-19 pandemic added to the burden of epilepsy itself.

The prevalence of depression in patients with epilepsy varied largely across the studies. In a meta-analysis of 51 cross-sectional studies published between 1999 and 2018 with sample sizes ranging from 36 to 1763, Yang et al. found that the prevalence of depression among patients with epilepsy (PWE) ranged greatly from 5.09 to 85.5% largely depending on the used diagnostic criteria [16].

The prevalence of depression in patients with epilepsy varied also during the pandemic. Sun et al. found that the prevalence of depression among PWE increased to 42.3% during the period of the pandemic [17]. Abokalawa et al. found that two-thirds of their sample of PWE (66.2%) reported depression during the pandemic [18]. Van Hees et al. found that 159/399 (39.8%) PWE scored positive for depression during the pandemic [19].

In our study, we found that a lot of factors related to the COVID-19 pandemic are significantly associated with increased frequency of moderately severe to severe depression. Patients who were afraid of COVID-19 infection, afraid of death from COVID-19 infection, had closed people infected or dead, had financial changes during the pandemic, had sleep disturbances during the pandemic, and continuously followed the pandemic news clearly suffered from more depression during the pandemic.

So, the COVID-19 pandemic is highly associated with increased prevalence and severity of depression among PWE which in turn has a severe burden on the patients.

These findings are consistent with other studies. Van Hees et al. reported increased depression severity with low financial status in PWE during the pandemic [19]. Sonbol et al. found that PWE who were concerned about the COVID-19 pandemic news and spent 3 h or more following the news had higher depression than the less concerned ones [20]. Kaya et al. reported increased depression levels in PWE if they encountered a COVID-19 patient or had a relative with COVID-19 [21]. Çilliler and Güven and Stauder et al. found that poor sleep quality was associated with higher depressive symptoms in PWE [22, 23].

In our study, there was a statistically significant association between increased depression severity and increased seizure rate, increased ER visits, decreased drug adherence, and decreased routine follow-up during the pandemic**.**

Our study is consistent with other studies who reported increased severity of depression in PWE is associated with decreased drug adherence in PWE during the pandemic [19, 24]. Other studies found that there was a significant relationship between increasing seizure frequency in PWE and increased depression severity during the pandemic [25, 26].

Depression itself is associated with depressed mood, lack of interest, and lack of activity that leads to decreased drug adherence which in turn leads to increased seizure frequency and increased rate of ER visits, so it is a bidirectional relationship. Our study is consistent with other studies who demonstrated a 2-way relation between a broad spectrum of psychiatric disorders (i.e., depression, anxiety, psychosis) and epilepsy [27, 28]. The bidirectional relationship suggests that common pathogenetic mechanisms are operant in both conditions, with the presence of one disorder potentially increasing the severity of the other [29]. Also, there is a decreased routine follow-up during the pandemic which in turn leads to worse control of the epilepsy.

Inconsistent with our results about the aspect of increased ER visits in PWE during the pandemic is Bamaga et al. [30] who found a 24% reduction in the number of ER visits for common neurological symptoms during the pandemic time in comparison to pre-pandemic. They explained this result by the public anxiety about the pandemic.

This inconsistency with our result can be explained by the fact that in our study 15.2% of the patients are illiterate and 54.1 % are secondarily educated. This lack of education makes it hard for the patient’s caretaker to fully understand how to support the patient during acute seizures and how to perform simple life-saving maneuvers like CPR which actually can be performed by many of the public in other communities. Moreover, this lack of education and low socioeconomic levels in our sample, along with the added financial problems during the pandemic, make it hard for these patients to access telemedicine services on the internet and mobile applications.

Using binary logistic regression analysis of the predictors of depression among the studied group during the pandemic, we found that these factors are significantly associated with increased frequency of depression in PWE during the pandemic: refractory seizures (more than 14-fold increase in the risk of depression, as a bidirectional link between depression and epilepsy), sleep disturbance (more than 12-fold increase in the risk of depression), low financial status (more than 11-fold increase in the risk of depression), increased seizures’ rate (7-fold increase in the risk of depression) (bidirectional relationship), being unemployed (5.8-fold increase in the risk of depression), increased ER visits (5.7-fold increase in the risk of depression), fear of COVID-19 infection (5-fold increase in the risk of depression), fear of death by COVID-19 infection (4.9 increase in the risk of depression), lack of drug adherence during the pandemic (4.2-fold increase in the risk of depression), decreased epilepsy-related follow-up consultations (3.7 increase in the risk of depression), and closed people death by COVID-19 infection (3.6 increase in the risk of depression).

Our results are consistent with other studies. Zis et al. found in their regression analysis model that unemployment and increased seizure frequency, in turn, increase the risk of depression in PWE [31]. Bifftu et al. found in their regression analysis model that perceived stress, seizure frequency of ≥ 1 per month, and difficulties of adherence to antiepileptic drugs were independently associated with depression in PWE [32]. Dos Santos Lunardi et al. found in their regression analysis model that increased seizure frequency, difficulties to access their physicians and anti-seizure drugs, and unemployment increase the risk of depression in PWE during the pandemic [24]. Yang et al. found that the most significant factors associated with an increased risk of depression in PWE during the pandemic were unemployment and poor antiepileptic drug (AED) adherence [16]. Our study is the first to assess a lot of predictors for depression in PWE during the pandemic, while the previously mentioned studies assessed only a few predictors that were consistent with our results.

The present study has some limitations; as the cross-sectional design of the study prevents causal conclusions, it just proves an association between depression with its determinants and COVID-19 pandemic and opens the floor for further longitudinal studies to prove causality. There are also several strengths of this study as it is the first study in Egypt that assessed the association between depression with its determinants and the COVID-19 pandemic and also the first study that examined a lot of determinants for depression in PWE during the COVID-19 pandemic.

## Conclusions

Our study revealed a high prevalence of depression in PWE during the COVID-19 pandemic. During the pandemic, patients with refractory seizures, sleep disturbance, low financial status, increased seizures’ rate, being unemployed, increased ER visits, fear of COVID-19 infection, fear of death by COVID-19 infection, lack of drug adherence, decreased epilepsy-related follow-up consultations, and closed people death by COVID-19 were the most significant predictors for depression in PWE. So, these risk factors must be evaluated and adjusted as this will be reflected in the improvement of the depression which in turn will be reflected in the improvement of epilepsy and on the quality of life of PWE.

## Limitations of the study

Our study has some limitations. Because the exposure and outcome are examined concurrently in a cross-sectional study, there is often no evidence of a causation link between exposure and outcome and longitudinal studies are recommended. However, we have many strengths in our study as our results are useful in focusing on PWE who are already under severe stress that increased more in the pandemic. Our study was performed by direct doctor–patient interview, not online or self-submitted questionnaires, which guarantees correct understanding of the patients to the questions and good interpretation of the results. Our study was performed in an epilepsy clinic and not in primary care clinic, allowing us to reach the medical records of the patients, which was very important to confirm the diagnosis, the type of seizures, the duration of illness, the number of anti-seizure medications, and the past medical history.

## Recommendations

We recommend that PWE should be regularly screened for depression especially during unusual circumstances like the COVID-19 pandemic. Early detection of depression in PWE and early adjustment of its risk factors helps for early treatment and better outcomes that will be reflected also on better management of epilepsy and better quality of life for those patients.

## Data Availability

All data generated or analyzed during this study are included in this published article.
